# "The Hospital" Nursing Mirror

**Published:** 1897-05-08

**Authors:** 


### "The Hospital" Nursing Mirror.

Being the Nursing Section of "The Hospital."

[Contributions for this Section of "The Hospital" should be addressed to the Editor, The Hospital, 28 & 29, Southampton Street, Strand, London, W.C., and should have the word "Nursing" plainly written in left-hand top corner of the envelope.]

### News from the Nursing World.

### THE QUEEN'S COMMEMORATION.

A meeting of the Executive Committee of the Queen's Commemoration Fund on behalf of the Jubilee Institute for Nurses was held last week at Grosvenor House, the Duke of Westminster presiding. It was announced that the Fund at present amounted to £50,465 16s. 10d., and it was decided to make renewed efforts both in London and in the country to increase this sum, having regard to the urgent necessity of more than doubling the total. Satisfactory reports of the progress of the Fund had been received from various parts of Scotland and Ireland.

### ENGLISH NURSES FOR BERMUDA.

We understand that the medical men in Bermuda are getting up a fund in celebration of the Queen's long reign which is to be devoted to the maintenance of two trained nurses. They are to be English nurses, and to be engaged on a three years' agreement. It is believed that their travelling expenses from London will be defrayed through private generosity.

### NURSING HOME FOR SALISBURY INFIRMARY.

It is stated that at a recent meeting of the Committee of Management of the Salisbury Infirmary plans for the erection of a Nurses' Home were adopted. The building will be in the shape of a T, and will have a south aspect, facing the Crane Bridge Road. The site is behind the Infirmary and between that building and the river Avon. The new Home is to be a three-storied building, containing about forty bed-rooms, and with good sitting-room accommodation. An appeal will be issued throughout the county for help, and will, we trust, meet with success.

### CHARITY BEGINS AT HOME.

A correspondence has been going on in the *Daily Mail* during the past week anent "Nurses for Greece," started by a medical man who has been, we think with great justice, much annoyed by the hasty withdrawal of a nurse in attendance upon a patient of his at a critical time in a serious illness in order to proceed to Greece. The nurse herself must be acquitted of blame in deserting her plain duties, for apparently she had no choice in the matter, being called away by the association to which she belonged. When nurses are needed in times of emergency there are ever plenty of the "unemployed" to be had, and there can be no sort of excuse for removing those who are in active and useful work at home. Why the nursing of a Greek soldier in a field hospital should be a "nobler cause" than that of saving a valuable life in an English home we fail to see. To "do the thing that's nearest, though it's dull at whiles" spells a far finer heroism than does the eager volunteering for anything which promises excitement and notoriety, now so much the fashion.

### A HINT TO HOUSEHOLDERS.

Lord Alwyne Compton, a member of the council of the Queen's Jubilee Institute for Nurses, has written to the papers suggesting that people who successfully let their houses or windows to sightseers for the forthcoming Jubilee Day might bestow upon the Commemoration Fund which is being raised in aid of the Institute five per cent. of the profits received. Thus they may properly "evince their loyalty by contributing . . . this small percentage towards a scheme which will assuredly render the name of Queen Victoria immortal among the poor for all time." Promises of support and subscriptions should be sent to the hon. secretaries, Mr. Harold Boulton and Mr. Ernest Flower, M.P., Queen's Commemoration Fund, 64, Cannon Street.

### TRAINED NURSES' CLUB.

We regret to hear that Mrs. Nichol, who has been for nearly thirteen years secretary of the Midwives' Institute and Trained Nurses' Club, has been obliged, in consequence of failing health, to resign her post. Mrs. Nichol has been connected with the club since its first beginnings, and she will be greatly missed. The council of the institute has placed the following resolution on the minutes: "The council of the Midwives' Institute have received with great regret the resignation of the secretary, Mrs. Nichol, who has for the past twelve and a half years filled the post. She has been greatly valued by the council and members for the obligingness, tact, and sympathy with which she has discharged her often difficult duties, and they feel that the club has lost a true friend. The council hope that rest will restore Mrs. Nichol's health, and that in the future they will often have the pleasure of seeing her at the club, to which they need not say she will always be a valued and welcome guest."

### HOSPITAL FOR EPILEPSY AND PARALYSIS.

A pleasant entertainment was got up last week for the patients at the Hospital for Epilepsy and Paralysis, Regent's Park, by the matron, Miss Miriam Ridley, and a number of her friends. Music and recitations were much appreciated by the audience, Messrs. Erard lending one of their pianos for the occasion. Several kind neighbours of the hospital lent glass and china and contributed flowers and plants by way of adornment. A very enjoyable evening was spent.

### THE "SWEATING" OF NURSES.

The pay given to district nurses in many parts of the country is extremely poor, and very often this is so, not because it is an actual impossibility to raise a few more pounds each year to maintain the nurse or nurses adequately, but because an idea exists on the part of some local committees that it looks well in their annual report to be able to state that Nurse So-and-So paid so many visits at the lowest possible cost. These good people do not appear to see that by this procedure they must be treating unfairly either the nurse, if they succeed in getting a fully-trained, competent woman for a very low salary, or the sick poor, if a half-trained, inefficient "nurse" is imposed upon them. So far from deserving commendation, therefore, this "cheap labour" business is utterly to be condemned on every ground. We have just seen a report of a meeting in a country district, at which it was stated that the "parish nurse" had done good work "at a cost of £32 10s. a year." Doubtless there must be some reason why the cost of maintenance in this instance is so abnormally low, but the special circumstances should be stated, otherwise a bald statement such as this is liable to lead to misconstruction, which may do great harm.

### GUARDIANS AND DISTRICT NURSES.

We are glad to see attention drawn by a correspondent to a contemporary to the fact that under an order of the Local Government Board dated January 27th, 1892, boards of guardians have the power to appoint district nurses. The writer goes on to say that "very little use has hitherto been made of this beneficent provision, chiefly because of the small amount of intelligent interest taken in such matters by the working people themselves," adding that "comparatively few people at present realise how much good can be done for the poor by a proper administration of the poor laws." Guardians, in their earnest endeavours to "guard" the rates rather than the sick poor, are only too prone to let this provision slip out of sight, and so far from appointing district nurses, usually sorely grudge the small subscription to existing district nursing institutions which is now and again suggested to them.

### DISTRICT NURSES FOR SHREWSBURY.

The Mayoress of Shrewsbury, Mrs. Cresswell Peele, presided recently over a meeting held at the Guildhall to consider a proposed "nursing scheme" in connection with the Queen's Commemoration at Shrewsbury. Mrs. Peele made an excellent speech, asking her audience to give not only their pecuniary aid, but also their hearty sympathy and approbation in founding a noble work in the city. She announced that donations to the amount of £435 9s. 6d. had been already received, and many promises of annual subscriptions. She had little doubt that if the ladies present would help her in forming a committee the whole sum would quickly be raised. Miss Baker, a nurse now working in Shrewsbury, gave an interesting account of the work being at present done. The scheme met with the universal approval of the meeting. Miss Gertrude Corbet was elected hon. secretary and treasurer of the committee. It is proposed to establish a home in affiliation with the Queen's Jubilee Institute, with a sufficient staff of nurses to provide efficiently for the needs of the city.

### NURSING AT THE BELFAST FEVER HOSPITAL.

The medical men attached to the Belfast Fever Hospital have sent in a report to the Board of Guardians requesting the appointment of five additional nurses, the present insufficient staff being assisted by pauper attendants, "too often the mere outcasts of society, and quite unsuited to undertake any duty in connection with the sick." The doctors state that, owing to the large number of cases in the hospital the nurses are overworked, while the patients are not receiving the personal attention and supervision which their condition requires. They add that they have good reason for stating that it is dangerous to the lives of such patients to be left in charge of untrained and irresponsible persons. A committee was appointed to confer with the doctors on the subject.

### LOCHWINNOCH PARISH NURSE ASSOCIATION.

The fourth annual report of the Lochwinnoch Branch of the Queen's Jubilee Institute for Nurses has just been issued, and records a good year's work. Nurse Sawyer paid over 3,000 visits during the twelve months ending March, 1897, the total number of cases on the books, including 21 from the previous year, being 209. The committee earnestly appeal for increased subscriptions and donations in order to avoid any trenching on the reserve of £200, which it is desired to keep for emergencies. Miss Hadden, district superntendent of Queen's Nurses, gave a satisfactory report of the work after an inspection in November.

### TRAINING AT FIR VALE INFIRMARY.

At a meeting of the Sheffield Board of Guardians, Dr. Martin, of Sheffield, gave his annual report in reference to his examination of the nurses of Fir Vale Infirmary in the theory and practice of nursing. Dr. Martin spoke highly of the standard of training, stating that most of the senior nurses were excellent in their proficiency, and that the junior nurses were progressing very creditably. This report must have given great satisfaction to the medical staff and to the lady superintendent (Mrs. Lawson), who are responsible for the establishment of this training school for infirmary nurses.

### A NEW NURSES' HOME.

The Works Committee of the Metropolitan Asylums Board have submitted plans for the erection of a new nurses' home at the Northern Fever Hospital, to provide accommodation for the assistant matron, night superintendent, and 98 nurses. The cost is estimated at about £17,000. The plans were approved by the Metropolitan Asylums Board, and will be forwarded to the Local Government Board for formal sanction.

### HAMMERSMITH DISTRICT NURSING HOME.

On Thursday H.R.H. Princess Louise, Marchioness of Lorne, presided at a meeting held in the studio of Mr. W. B. Richmond, R.A., Beavor Lodge, Hammersmith, to promote the formation of a Women's Jubilee Memorial Fund in aid of the Hammersmith and Fulham District Nursing Home. Mrs. Garrett Anderson, Mrs. Dacre Craven, and Mrs. Roland Hunt were among the speakers.

### SHORT ITEMS.

A nursing home is to be established in Slough as a permanent memorial of the Queen's Record Reign.—A revised set of rules for the working of the Lady Wenlock Nursing Institute in connection with the Madras General Hospital, originally organised by Mrs. Nesbit, the lady superintendent, has been drawn up and provisionally approved by the Government of India.—Mrs. Bedford Fenwick has, we understand, volunteered to take charge of the nurses who have already left England for the Greek frontier, and started for this purpose on Friday last. Before her departure, Mrs. Fenwick was honoured by being received at Marlborough House by the Princess of Wales, who has evinced much interest in the preparations for ending aid to the Greek wounded.—Messrs. Garrould, Edgware Road, ask us to state that they have forwarded to the Crown Princess of Greece, through the "*Daily Chronicle* Fund," a large supply of clothing for the use of the Greek wounded.

### Pharmacy and Dispensing for Nurses.

By C. J. S. THOMPSON.

### IX.—DISPENSING DIFFICULTIES—INCOMPATIBLE MIXTURES.

OF the many difficulties that may present themselves from time to time to the dispenser we can only mention a few, but they will serve to illustrate the fact that, with a little fore-thought and skill, an unsightly appearance in many compounds may be avoided.

The preparations of iron are a fruitful source of trouble. They are incompatible with alkalies and their carbonates, and also turn black or darken all vegetable astringents that contain tannic acid. In preparing a mixture of solutions of dialysed iron and arsenic, with glycerine and water, care must be taken not to add the Fowler's solution of arsenic until the iron has been well diluted with water, otherwise a precipitate will be formed. When powdered gum tragacanth is met with in a mixture, advantage should be taken of any alcoholic liquids ordered in the prescription to mix them with the gum before adding the water, when by vigorous shaking a mucilage can easily be formed. Quinine is incompatible with all alkalies and their carbonates, and iodides. When mixed with vegetable infusions containing tannic acid, a precipitate of tannate of quinine is thrown down. Quinine is readily soluble in acids, tincture of perchloride of iron, and aromatic spirit of ammonia, but is precipitated from the latter on the addition of water.

In the following prescription the whole of the menthol (if the tincture of iodine be added to it and the glycerine) will collect and form a cake on the top of the liquid. ℞. Tr. iodi. ʒv, menthol ʒiss, glycerine ℥i, aquam ad. ℥iv. This may be prevented if prepared in the following manner: Dissolve the iodine and potassium iodide (composing the tincture) in the water, then powder the menthol and rub it up with the glycerine, adding the rectified spirit of the tincture last of all.

In the following mixture decomposition takes place owing to the action of the spirit of nitrous ether on iodide of potassium: ℞. Potass. iodid., ʒiss.; tr. digitalis, ♏xxx.; spt. ether nitros, ʒvi.; syr. simplx., ℥i.; aq. ad., ℥viii. Misce. This may be prevented by neutralising the spirit of nitrous ether with a little bicarbonate of potass before adding it to the mixture.

Quinine and salicylate of soda when ordered together in a mixture are a frequent source of difficulty, as instanced in the following: ℞. Quinæ. sulph., grs. xii.; acid. hydro-brom., dil., ʒiss.; sodæ salicylas, ℈ii.; syr. simpl. ʒvi.; aquæ ad., ℥vi. Misce. In preparing this mixture the quinine sulphate should first be dissolved in the hydrobromic acid and diluted with three ounces of water and the syrup. Then dissolve the salicylate of soda in the remainder of the water, and add slowly to the previous solution, shaking gently between each addition.

The following prescription cannot be properly dispensed as written, owing to the bicarbonate of sodium causing the precipitation of the cocaine: ℞. Sol. cocainæ hydrochlor., 2 per cent.; sodii. bicarb., ʒss.; glycer. boracis, ʒi.; ac. carbol., ♏v.; aquæ, ℥i. Misce. If ten grains of sodium chloride be used in place of the sodium bicarbonate the reaction is prevented.

In the next example a double reaction takes place, resulting in the formation of insoluble calcium sulphate and iron hypophosphite. This cannot be avoided. ℞. Potass. chlorat., ʒss.; calci. hypophosph., ʒss.; magnes. sulph., ʒi.; ferri. sulph., grs. xii.; liq. strychnine, ♏xii.; aquæ, ℥vi. Misce. In incompatible mixtures of this class the dispenser must endeavour to prepare them in as presentable a condition as possible.

Perchloride of mercury is incompatible with mucilage of acacia, albumen, and gelatin, forming insoluble masses. With iodide of potassium a precipitate of iodide of mercury is thrown down, which must always be carefully avoided in dispensing. It should never be brought into contact with metals, especially in the presence of moisture, and the student must beware of using a damp palette-knife with this salt. When dispensing the perchloride with mucilage, the former must always be well diluted with water before adding the latter, or an unmanageable mass will be produced. The subjoined prescription illustrates a mixture of this kind. ℞. Hydrarg. perchlor., grs. iv.; mucil. acacia, ʒiv.; spt. chlorof., ʒii.; liq. potass., ʒi.; aquæ ad., ℥iii. Misce. In dispensing this, the perchloride of mercury should be dissolved in an ounce of the water, and the mucilage may then be added. Dilute the solution of potass with the remainder of the water, mix the solutions, and finally add the spirit of chloroform.

It will be well to bear in mind the following list of incompatible substances. The vegetable alkaloids are nearly all precipitated by tannic acid. Acacia mucilage is incompatible with alcohol, sulphuric acid, borax, perchloride of mercury, persalts of iron, and subacetate of lead renders it gelatinous; acid gallic with spirit of nitrous ether, and metallic salts; acid hydrochloric with salts of silver and lead, tartarated antimony, and alkalies; acid nitric with alcohol, alkalies, oxides, sulphate of iron, acetate of lead, and carbonates; acid tannic with mineral acids, alkalies, salts of antimony, lead, and silver, persalts of iron, vegetable alkaloids, and gelatine; ammonia carbonate with acids and acidulous salts; tartarated antimony with gallic and tannic acids, the alkalies, lead salts, and astringent infusions; antipyrine with hydrocyanic acid, tannic acid, butyl chloral hydrate, chloral hydrate in strong solutions, astringent infusions, nitrites in solution, and astringent tinctures; salicylate of soda, when mixed together in powder, and when combined with spirit of nitrous ether, turns it a green colour; bismuth subnitrate with potass, soda, and ammonia, and their carbonates; cinchona preparations, with ammonia, antipyrine, metallic salts, and gelatine; digitalis preparations with sulphate of iron, tincture of perchloride of iron, preparations of cinchona and acetate of lead; iron and ammonia citrate with mineral acids, vegetable astringents, and fixed alkalies; iron and quinine citrate with alkalies and their carbonates, tannic acid, and vegetable astringents; iron perchloride with alkalies and their carbonates, magnesia, and its carbonate, vegetable astringents blacken it, and all vegetable infusions are darkened in colour with the exception of quassia and calumba; mercury perchloride with alkalies and their carbonates, tartarated antimony, nitrate of silver, acetate of lead, albumen, iodide of potassium, soap, and decoction of cinchona; mercury subchloride (calomel) with alkalies and their carbonates, sulphides, hydrocyanic acid, bitter almonds, lime water, iodide of potassium, iodine, nitric acid, salts of iron, lead, copper, nitrate of silver, and soap. Soap should never be used as an excipient for pills containing calomel. Magnesium sulphate with alkaline carbonates, and acetate of lead; morphine hydrochlorate with alkalies, astringent infusions and decoctions; opium preparations with alkaline carbonates, salts of lead, iron, copper, and zinc, solution of arsenic, and vegetable astringents; subacetate of lead solution with hard water, mineral acids and their salts, vegetable acids, iodide of potass, all astringents, preparations of opium, and albuminous liquids; potassium iodide with bismuth subnitrate, spirit of nitrous ether, decoction of liquorice, preparations containing starch or acid, and vegetable alkaloids; potassium permanganate decomposes when mixed with organic bodies; quinine with all alkalies and their carbonates, astringent infusions, salicylic acid and its salts; infusion of roses with alkalies and borax turns green in colour; spirit of nitrous ether with antipyrine, iodide of potassium, tincture of guaiacum, sulphate of iron, gallic and tannic acids.

### Post-Graduate Clinics for Nurses.

By a Trained Nurse.

XII.—THE DOMESTIC ECONOMY OF ILLNESS (*continued*).

The domestic economy of illness has so distinct and practical a bearing on the life and work of a private nurse, and opens up so many interesting possibilities that it may be well to devote the space of this week to some more points towards which the nurse may with advantage direct her housewifely and thrifty gifts. Most of us remember the injunctions laid upon us when we were in Hospital to "be careful of the linen," and recall how Sister used to exercise a watchful eye over a nurse endowed with æsthetic aspirations towards beauty and cleanliness lest her artistic standard might blur her discretion as to the proportions of the Hospital laundry account.

In private practice the nurse will often be accorded considerable scope for lavishness in laundry matters, and she will not usually have to husband with economical foresight her stock of linen, or have recourse to the masterly methods exhibited by some nurses in Hospital who so dispose of a clean sheet or a fresh pillow-case as to give the effect of clean linen throughout the ward. Many patients in private practice are sufficiently prosperous to give *carte blanche* to a nurse in the matter of dainty nightgowns, frilled pillow slips, and unlimited clean sheets. But the majority of mortals are not so favoured in the matter of income, and in these cases the nurse, while aiming at keeping her patient as fresh and attractive-looking as may be, must bear in mind the state of the family finances, already, perhaps, seriously encumbered with the hundred and one additional expenses following as a matter of course in the trail of an illness. A London laundry bill, unless skilfully engineered, has a tendency to mount up in a manner threatening to a limited banking account. And it is surprising what a careful nurse may do in the direction of economy of bed and body linen by alternating sheets, airing and folding them very neatly, and using them again. In many houses, too, a linen press is kept for smoothing out table-cloths and serviettes. The housemaid can usually be induced to pass sheets and pillow slips—crumpled, not soiled—through these, the bed thereby looking as fresh and nice as if clean sheets were put on daily. Economy of tray-cloths and serviettes may be observed without any detraction from the daintiness which should always be observed in the dietary of the sick-room. I have seen many a thrifty nurse who has found by experience that her patient has a habit of spilling gravy or particles of food on his tray-cloth each time a small repast is served, thus making a clean cloth necessary for every meal substitute a decorative dish-paper for the linen tray-cloth, thus providing a dozen pretty coverings for the tray for the same price as the single washing of a linen cloth. On the other hand, I have seen nurses, born without the talent of domesticity, and whose training has evolved no knowledge of domestic economy, use fresh clean serviettes either as dusters or for wiping up water spilled; and in some few exaggerated instances I have observed them shocking some good housekeeper by deliberately mopping up a medicine—even an iron tonic—with a fine dinner napkin. To spill lotions and iron mixtures on nightgowns and sheets is by no means an unknown circumstance in the annals of private nursing, and the crop of "ironmould" returning from the weekly wash is not calculated to inspire the domesticated "lady of the house" with a fervent affection or admiration for the trained nurse. I recall now an instance of a nurse whose theories of disinfecting linen were much more admirable than her practice. She was nursing a small boy suffering from measles whose mother, with more pride than practicality, endowed his bed with beautiful new blankets and much fine linen. At the conclusion of the illness the certificated nurse in attendance volunteered to disinfect the new blankets. With every good intention, and with a knowledge of the general excellence of Condy's Fluid as a disinfectant, she selected this among so many others more suitable to bedding, and made such a strong solution and mixed it so inefficiently that the "beautiful new blankets" came out of their Condy tub with huge, deadly-looking patches over their entire length and breadth. And these spots, like those of the leopard, refuse to change, and remain to this day as a dark monument to the valuelessness of theory and the advantages of a practical domesticism in nursing. Again, I have met with instances of sheets absolutely ruined by their immersion for purposes of disinfection in a solution of perchloride of mercury, than which nothing can be more admirable for every antiseptic purpose—but that of linen. This it discolours and hardens beyond all redemption. It might be advisable to remark at this point that pure perchloride of mercury should not be poured down w.c.'s on any pretext or for any purpose whatever. I mention this because a few days since I saw a firm of plumbers in paroxysms of excitement over the havoc wrought in some drain pipes by the heroic determination of a nurse not to propagate typhoid by distributing untreated discharges through the public sewers. This is another instance of theory waging war against common, every-day knowledge. Among the many much-needed additions to the practicalities of the Training School is the instruction of the probationer from the first day she enters the Hospital as to the market value of every appliance, surgical or medical, which is likely to be used in the wards or in any sick-room. I would like to see price-lists, legibly printed, put into the hands of every probationer, and posted up in all nurses' sitting rooms, setting forth the price per yard of every gauze, dressing, and bandage used throughout the Hospital. Were she familiar with prices, no nurse would allow patients to use white water bandages for garters, nor would she sacrifice lint for the washing of the sick. Recognising the strain on the Hospital resources occasioned by apportioning a quarter of a yard of lint to nearly every patient in a ward for "wash-rags," she would invent some other expedient. In the days of my training, I was taught by the charge nurses of one of the largest and best training schools in London that this appropriation of lint was legitimate and proper. Had I for one moment realised the market value of lint, I should have hesitated seriously before adopting this custom then universal in the Hospital. In justice to the nurses, it is fair to point out that the authorities provided no other means whereby we might wash and scrub the skins of our patients, often grimy enough. "Tow," of course, served for the first grand wash when the patient was admitted—lint afterwards was utilised for this purpose. I hope some day to see so fine a training for nurses established that every form of object-lesson and real practical teaching will take the place of so much which at present is purely "booky" and theoretical. The private nurse would find it much to her advantage were she more or less of a walking price list of invalid appliances, and it would not then be possible to quote such an instance as the one I bear in mind where a private nurse used iodine for the disinfection of typhoid fever discharges. She explained that "iodine was so effectual"—in which she was perfectly correct. But she was totally unaware, till she saw the resultant bill for "drugs supplied," that the price of iodine does not warrant its use where the crudest and cheapest disinfectant, such as sulphate of zinc, would be equally effectual.

### Nurses on Wheels.

Running westward with the Loire from Orleans to the sea is one of the most beautiful highroads of France. The general idea of our tour was to follow this road to Nantes and then strike off by road or rail to Les Sables d'Olonne, "La plus belle place de l'Europe." On this tour we had decided to wear English skirts and send on luggage from day to day, French railway arrangements allowing luggage to be sent on by Grande Vitesse for a very small sum. However, in future we should certainly discard skirts when riding abroad, and using ordinary men's bicycles should carry enough luggage for at least two nights with skirts strapped to the handle bars. However convenient railway arrangements may be, wind or weather sometimes make it disagreeable to have to reach the place decided upon.

We left Orleans about ten in the morning, crossing the Loire to the south bank by a great bridge—alas! not the famous one sacred to the memory of Jean d'Arc, for that has long since vanished. About a mile on the way to Blois we saw a vineyard for the first time. It was a bitter disappointment. I had pictured a waving mass of vines growing high overhead in interlacing branches, the ripe fruit hanging down in great bunches. Perhaps the fable of "The Fox and the Grapes" was responsible for this idea, but certainly the smallest fox could have no difficulty in reaching these vines, which grew in rows no higher than healthy potatoes. However, in this case the grapes were really too sour even to steal, for they are not ripe until October, and this was August. Our first stop was at Cléry, with its stone-flagged single street of houses and beautiful old church. Everything seemed asleep in the silence of noonday. We lunched at the little inn, and afterwards remembering the devotion of Louis XI. to the famous black virgin of Cléry, we entered the church and bought a little leaden image, the exact duplicate of that which Sir Walter Scott describes him as wearing in the rim of his weather-beaten, unkingly hat. Wheeling our bicycles over the cobbles (a precaution which later on we forebore, as every village on the high road has its own length of cobbles), we struck off southwards to visit the great Chateau of Chambord, which lies a few miles to the south of the river. This was the Versailles of Touraine until Louis XIV. moved the Court nearer to Paris. It was plundered during the Revolution, but was restored by the Government, and its great mass of towers and pinnacles are almost in their original condition. The interior, however, is somewhat bare. Leaving Chambord we rejoined the Loire a few miles before Blois, which is built on a steep hill crowned by a beautiful castle. We had time before dinner at the Hotel du Chateau to climb up and down some of the streets of stairs that run straggling up the hill.

Early next morning we explored the castle, the famous spiral staircase leading us into the suite of apartments wherein the tragedy of the Guises was consummated. All these rooms have been gorgeously redecorated lately, and the walls are hung with canvas, painted in vivid colours. We saw the spot where the murder took place, and the little room in which prayers were offered up for its success. In the afternoon we rode on to Amboise, the road now running between the river and the cliffs, and we passed a great many of the cave-dwellings so common in this part of France. The natural caves are bricked up in front, and windows let in. Some of them make very comfortable little homes, and even have pretty gardens in front. Many of these caves were used as hiding places in sturdier times, and many a Huguenot took refuge in them. The castle at Amboise is situated high up on the cliff, but, as restorations were going on, we could not go inside. We walked about the terrace and saw the balcony from whence the whole Court, including Mary Stuart, was made to witness the drowning of the Huguenots in the river below by order of the Guises. The garden is lovely. At one end of the terrace weird trees, deftly interlaced, form a complete cover from the wind or sun, and further on is the exquisite little chapel of Francis I., which Ruskin has written so eulogistically about. Our hotel at Amboise was the Cheval Blanc, very primitive, but clean and absurdly cheap. We made an early start next morning as our destination was Chinon, and there was much to be seen *en route*. At Vouvray we just stopped for lunch and then rode on to Tours, where we spent about two hours wandering about the town and splendid cathedral.

About half an hour's journey by train took us by Langeais. The chief interest and beauty of this sleepy little place is centred in the castle, which now belongs to M. de Sigfried. The interior is most beautiful, all the old tapestries being in good preservation, and the rooms, although in use, carefully kept in their original condition and arrangement. Anne de Bretagne was married in the chapel here to Charles VIII. Leaving the Loire to the north, we rode on to Azay le Rideau, with its sixteenth-century castle on the Indre. After Azay the road to Chinon was very bad; it runs through a tract of forest, and is chiefly composed of sharp flints embedded in loose sand. We were now in the so-called Valley of Rabelais, and arrived at the little River Vieuve and Chinon at the same time.

It was too dark to see anything of Chinon that night, so we went straight to the Hotel de France, which we found exceedingly comfortable. Rabelais lived at Chinon for some years, and a bronze statue has recently been set up by the riverside to his memory. The Castle is the mere husk of a castle, but the four walls are standing of the grande salle in which Jean d'Arc was first presented to the King. We rode to Saumur in the afternoon, a distance of about nineteen miles, through orchards, walnut groves, and vineyards, passing by the farm in which Rabelais was born. Leaving our bicycles at the Hotel Budan we walked out two miles to the south of Saumur to see the Great Dolmen of Bayeux, the largest Druidic remains in France. The Castle at Saumur is built upon a high ridge, and commands a splendid view, and the walls are deeply indented by the bullets of the Vendéans, as also are the walls of the Hotel de Ville. Only two towers and a prison house now remain of the old fortifications. After déjeûner we crossed the bridge on our way to Angers, which lies about 30 miles down the river. It rained and blew hard against us all the way, and I punctured my front tyre when far from shelter, so that Angers will always be associated in my mind with considerable discomfort, an impression, however, which the Hotel de Cheval Blanc did its best to dispel. Henry III. apparently bore no good will to Angers, for, by his order, the 17 towers encircling the castle were all decapitated and levelled with the rest of the structure. We went next day to Nantes, which was to be the end of our tour on wheels. The first part of the road outside Angers was very bad going, owing to the soft surface of powdered coal, but after St. George the road is good all the way. At Champtocé we turned aside to see the ruins of the Chateau of the original Blue Beard. He was a Sieur de Retz of the time of Charles VII., who is reported to have bathed in the blood of children as a magic cure for his ill-health, and it seems that many a child was decoyed within the grim walls in order to provide the daily bath. He paid the penalty for his misdeeds, for he was eventually put to death as a sorcerer. The road now rolls on past Encenis with its splendid suspension bridge, and Chamtogeaux, where the unfortunate Jean de Montfort was so long imprisoned, and then the great town of Nantes came in sight, and a long run down hill brought us to the first of the four bridges we had to cross before arriving at the Hotel de Bretagne, and the end of our tour on wheels.

### Queen Victoria's Nurses.

The following verses by the Very Rev. the Dean of Rochester appear in the current number of the *Ladies' Realm*:—

"Queens shall be thy nursing mothers."
How shall we teach our children to proclaim
With love like ours Victoria's worth and fame?
And how to future generations show
Those works of mercy all her people know?

Shall the historian, shall the poet's song,
Those deeds perpetuate, and that praise prolong?
Shall art on canvas or in sculpture trace
For aye the records of that royal grace?

Brief while such annals in men's minds remain,
Years dim the picture, and the marble stain;
Whence shall the living presence then appear,
To move and speak, for all to see and hear.

She whom we seek to honour shall reply;
Her works unto her wish shall testify;—
What was her choice, and what the end she sought
With all that gold her subject sisters brought? *

Queen though she was, Christ's servant; and His zeal,
The poor to comfort, and the sick to heal,
Glowed in her heart; and at her sweet command
"Victoria's Nurses" came to bless the land—

To lonely cottage and to crowded street,
Where pain and nakedness and hunger meet,
With sure relief unto the sore oppress'd,
Strength to the weak, and to the weary rest.

As yet but few, though all who know commend,—
Shall we not seek their mission to extend?
Till thousand thousands bless the day they came,
Those royal nurses in Victoria's name.

So through the ages that dear name shall tell,
To poor sick folks, from one who loved them well,
Bright words of hope, such as an angel brings,
With God's own glory on his golden wings.

* An offering of £70,000 from the women of England, in commemoration of Her Majesty's Jubilee in 1887.

### Minor Appointments.

Newcastle-on-Tyne Union Hospital.—Miss Elsie Wake has been appointed Night Superintendent at this institution. Miss Wake received her training at the Newcastle Royal Infirmary, afterwards being promoted to the post of head nurse at that hospital. Subsequently she took up private nursing, and, after three and a half years, returned to the Royal Infirmary, where she has worked as nurse and head nurse till the present spring.

Chichester Workhouse.—Miss Mabel Hamerton has been chosen to fill the post of Superintendent Nurse at the Chichester Workhouse. Miss Hamerton was trained at the Deaconesses' Institute, Tottenham, and has subsequently held appointments as superintendent sister at the British Lying-in Hospital, and at the English Hospital, Jerusalem.

Suffolk General Hospital, Bury St. Edmunds.—Miss Mary Bratton has been selected to fill the post of Night Sister at this institution. Miss Bratton is a Nightingale nurse, and was for three years at the Marylebone Infirmary, subsequently holding an appointment as sister at Rotherham Hospital for rather more than two years.

The New Hospital, Gwelo, South Africa.—Miss Henrietta Kenealy and Miss May Muriel have been appointed Sisters at this hospital. Miss Kenealy and Miss Muriel both hold the certificate of St. Bartholomew's Hospital.

### Appointments.

MATRONS.

The New Hospital, Gwelo, South Africa.—The British South African Company have enlisted the services of Miss Mary Hood Harding as Matron for their new hospital at Gwelo, on the borders of Matabeleland. Miss Harding received her training at King's College Hospital, and has since held the posts of sister at Pendlebury for three years, and matron of the Jenny Lind Infirmary, Norwich, for over two years. Miss Harding, accompanied by Miss Henrietta Kenealy and Miss Mary Muriel, who have been appointed sisters at the hospital, left Southampton on May 1st. They take with them very many good wishes for their success and happiness in the new life awaiting them.

Chelmsford and Essex Infirmary and Dispensary.—Miss Martha Cash has been appointed Matron at this institution. Miss Cash received her training at the Halifax Royal Hospital, and has subsequently held appointments at the Hospital, Stafford, the Infirmary, Burton-on-Trent, and at Stockport Infirmary. This latter institution she now leaves to take up her new work, and she takes with her many good wishes for future success.

### Presentations.

Miss Adelaide Griffiths, upon leaving St. George's Infirmary (Hanover Square Union) to be married, was presented by the nurses and staff with a chest of silver-plated table cutlery; also by Dr. Webster (medical superintendent) with a chest containing silver-plated and pearl dessert and fish knives and forks. Much regret is felt at Miss Griffiths' departure. She has held the post of matron at St. George's Infirmary for the last eighteen months, and during that time has gained much esteem from guardians, patients, and staff.

### Death in our Ranks.

We regret to announce the death of Mrs. Davies, out-patient nurse in the ophthalmic department at St. Thomas's Hospital, on April 17th, after a very short illness. She was buried at Norwood Cemetery. The first part of the funeral service was held in the hospital chapel, and was attended by many of the staff and a large number of nurses. The coffin was covered with lovely wreaths and flowers, sent by the ophthalmic visiting surgeon, the resident staff, fellow nurses, and other friends. Nurse Davies entered the Nightingale Fund Training School in July, 1874, and in September, 1875, went to the Edinburgh Royal Infirmary. She returned to St. Thomas's Hospital in December, 1882, and was appointed out-patient nurse to the ophthalmic department, which post she held until her death. As she was connected for so long a time with the hospital, and was well known to many old Nightingale nurses, she will be much missed both by them and also by those who are at present in the hospital.

### Wants and Workers.

Nurse Jarvis, The Union Infirmary, Hitchin, Herts, wishes to know whether any kind person will give her a reclining or cane deck chair for the use of her paralysed patients who, from the want of such an article, are confined to their wards all the summer days.

The Hon. Secretary of The Hospital Convalescent Fund acknowledges, with grateful thanks, contributions in response to the appeal in last week's issue for "Nurse K.'s" travelling expenses to America from the Hon. Sydney Holland, "A Reader in Inverness," "Three Nurses," "Sister G.," E. H. Cordner, E. F. N., Nurse Birdseye, Nurse M. G., "A Nurse," and Nurse E. Duckett. These kind friends have made up the required sum, so that Nurse K. will be able at once to complete arrangements for her journey. The Hon. Secretary will have much pleasure in conveying to her the many kindly wishes for her recovery, and warm expressions of sympathy which have accompanied the contributions.

### The Nursing of Phthisical Patients.

By Louis Vintras, M.D.Durh., B.Sc.Paris, Licentiate of the Royal College of Physicians, Physician to the French Hosp tal.

There is, perhaps, no disease which taxes the resources of a nurse so much as phthisis; the generally long duration of the malady, the character of the patient, the sudden and unexpected changes, the particularities of the diet, the continuous attention to material cares, the necessity for a constantly lively disposition in the midst of most depressing surroundings, are so many causes which strain to the utmost the powers of resistance of those called upon to be in constant attendance on such cases. But if the task is arduous the results are generally most gratifying, for these patients are particularly sensible to even the smallest cares, and the effect of careful nursing is bound to show itself almost immediately in their more contented—often distinctly happier—appearance.

In choosing a room for a consumptive patient, the great point to bear in mind is that it should be bright. The aspect should be southern or western, overlooking a garden, or, if this is out of the question, the street. Rooms giving on courts or facing blank walls, as is too often the case even in the country, are most depressing, and, as such, should be studiously avoided. For with those suffering from this disease, quietness is not so important as brightness; they require to see as much life around them as possible, and since their condition when confined to their rooms precludes their having movement and life in their immediate surroundings, they should be able to find it as near as possible. The patient who can look out on the world from his window has already a host of little interests in existence, and to interest a consumptive person in existence is the surest way of warding off that intense melancholia which saps his mental energies as surely as the disease itself saps the vitality of his body.

The room should be brightly furnished, well lit, well ventilated, well warmed, and the atmosphere should be kept as dry as possible. As far as convenient medicine bottles and nursing necessaries should be placed out of sight; and if a receptacle is required for the expectoration, it should be some elegant little vase, which should be emptied frequently and well cleansed, thus doing away with the necessity of leaving in it an antiseptic solution, which is often nauseous. In fact, everything which tends to remind the patient of his condition should be carefully avoided.

The room should not be overheated, as this of itself will cause the patient to cough, and in phthisis everything should be done to check the cough. In fact, much may be realised in this direction by the patient himself, if he is shown the importance of controlling the cough, which is frequently partly nervous. There are certain institutions in Germany for these cases, where much stress is put on this point, with the result that, after a time, excessive coughing and expectoration is looked upon by the sufferers themselves as not quite *comme il faut*.

If the night-sweats are profuse, the patient should sleep between blankets or flannel sheets, as the continued changing which otherwise often becomes necessary is most fatiguing and detrimental.

The temperature is most important and should be taken regularly, though it may remain normal for weeks, as any rise is indicative of rekindled trouble in the lung, and any fall below normal of depression and loss of strength. By being early recognised, both may be checked; while after a few days, it may require long weeks to bring about the same result.

Next in importance to the temperature is the weight of the patient; a consumptive person who puts on flesh is recovering. This may be taken as an almost infallible test, however slow the process may be, and however little the gain. The patient should therefore be weighed regularly once or twice a week and a record of the weight kept.

Great attention to the digestive functions is also necessary. When up, the patient should always wear flannel or woollen underclothing. The clothes should be warm and light, as heavy things only fatigue. When able to go out, in this climate at least, it is better for him to be moving, either walking or driving. He should return home before feeling tired, and a short outing twice a day is better than a protracted one.

Any reference to the disease itself should be avoided in conversation, and the patient should be prevented, as far as possible, from brooding over his condition. His mind should be constantly occupied, and reading, music, and games, when possible, will all help to this end. Visitors in reason should also be encouraged.

Persons suffering from phthisis are often peevish, irritable, and at times vexatious; a gentle manner, kind words, and a bright face will do more to soothe them than any uncalled-for coercion. Anything to which the patient shows a marked objection is better left aside for a time. Even in the most favourable cases long months must elapse before any real result is obtained, and it is but natural that now and again the patient should become weary, discouraged, discontented. The nurse who is constantly by his side can do more to restore his hopes, to show him what he has already gained, than the physician whose visits are necessarily limited.

On the question of food, individual tastes should be studied, avoiding especially the common error of forcing heavy meats on a delicate stomach. Pork and veal are most injurious, and it should be remembered that beef takes a long time to digest as its fibres are coarse, and though most sustaining in health, it is apt to irritate the digestive organs of these weak patients, and remain undigested. The food should be nicely served up, taken frequently, and varied as much as possible. Respecting beverages, it is necessary to avoid over-stimulating the patient, and the choice of wines is a most important one when the dyspepsia is advanced, as certain red wines are apt to turn acid on the stomach. On this score, however, I must confine myself to such generalities, as otherwise I should be trespassing on the subject of the treatment of phthisis.

The choice of climate is also hardly relevant, but it may be mentioned in passing that the general belief that sea air is beneficial to the majority of these cases is a dangerous fallacy.

In the above remarks I have only been able to indicate the spirit in which the nursing of these cases should be undertaken; but I have said enough, I think, to show how different are the exigencies of this disease compared with others. The number of victims which phthisis claims each year in London alone is warrant enough for the nursing community to devote special attention to this disease. Here more than elsewhere the nurse has to be the friend of the sufferer, and the more cultivated her mind is the more precious will she be. It would certainly be a great advantage if a class of nurses were instituted who would follow a special preliminary course of lectures and who would devote themselves to these cases, and here those who have the advantage of a refined education would find a natural channel for the blending of their past acquirements with their scientific training. For nurses used to the hard and fast lines of acute medical or of surgical cases could hardly be expected to succeed with those suffering from this tedious malady with its ever-changing phases. A phthisical patient is a study in psychology, and as such requires on the part of his attendants a considerable amount of ingenuity. Again, from the very nature of the disease, the nurse is often thrown for long periods entirely on her own resources, and much will depend upon her experience and the habit she has acquired of dealing with such cases. Such a suggestion is, I fear, rather ambitious, but it is one which may some day find its application, when the great army of nurses, now established, has had time to settle down and consider the best modes of employing its varied energies.

### Everybody's Opinion.

[Correspondence on all subjects is invited, but we cannot in any way be responsible for the opinions expressed by our correspondents. No communication can be entertained if the name and address of the correspondent is not given, or unless one side of the paper only is written on.]

### NURSES' UNIFORMS.

"QUEEN'S NURSE" writes: Ought we to get up a petition praying for the protection of our uniforms? I most earnestly wish some steps could be taken to prevent nursemaids and others from copying uniforms worn by "accredited nurses." I am district nurse and never think of wearing uniform except when on duty, because I am so disgusted to see the way in which it is copied by those who have no right to wear it. I should not think of wearing a nun's dress or dressing up as a widow. Why should our dress be copied by servant girls? Cannot someone do something to get up a petition? I was very glad to see the suggestion made in THE HOSPITAL two weeks ago.

### A HINT TO NIGHT NURSES.

"L. W." writes: Kindly allow me through the medium of your widely-read columns to send a message to those nurses who, like myself, have suffered from inability to sleep during the day when on night duty. After having tried every imaginable thing to induce sleep in vain, I have at last hit upon a plan which is a complete success. On going to bed I open the window a few inches at the top and cover it up with a thick rug, blanket, or quilt, so as to exclude every ray of light, making the room perfectly dark. By taking these simple measures I find I am able to sleep as well in the day as at night, and I think my discovery may be useful to others who have the same difficulty to contend against, as secure to weary night nurses hours of refreshing sleep.

### ALMSHOUSES FOR NURSES.

"QUEEN'S NURSE" writes: I have read with much interest the letters *re* "Almshouses for Nurses"; also the one headed "Nurses' Earnings." A house or houses of rest would be good, so would an increase of salary. But I think that the best thing that the Queen's Jubilee Institute could do for her nurses would be to establish a pension fund for Queen's nurses, and failing this the next best thing would be for the Queen's Institute to join the Royal National Pension Fund on behalf of Queen's nurses, the same as some of the good hospital committees have done for their nurses. Many Queen's nurses have to live in separate lodgings, which is expensive, be you ever so economical, so much so that they cannot well join the Pension Fund for themselves, at least not with our present rate of salary.

"A SISTER" writes: I have read with much interest the discussion regarding nurses' uniform, and although it would I think be nice to have it registered, yet Nurse H.'s remarks savour of unoriginality, as at present it appears an almost impracticable thing. Might I, therefore, suggest, or rather give an opinion that I have held for some time, that hospitals, institutions, &c., requiring their nurses to wear a uniform should supply them with a uniform coat and skirt and sailor hat with the arms or badge of the hospital stamped on it, in place of the hackneyed cloak and bonnet. This, of course, would not be so attractive or conspicuous as the present nurses' (?) uniform, and I am convinced from experience that nurses would not find it took much longer to put these on than a cloak and bonnet, especially as it is now a recognised fact that nurses should not wear the same dress outdoors as in the wards. I feel myself this would exclude a great many hypocrites from wearing a uniform and disgracing it.

"A MATRON" writes: I quite agree with the writer of the letter about almshouses for nurses. It is a provision which is greatly needed, and it would be a real boon to many nurses who are confronted with the problem of how to find a home for their old age when work days are over—a problem which, so far, has not found any satisfactory solution. I was much struck in reading an account of Pastor Fliedner's Institute at Kaiserworth with the excellent provision made there for the nurses when too old for duty. During all their years of active service they have the comfort and help of being able to look forward to spending the evening of their life in the Home for Aged Deaconesses—an institution forming part of the hospital where they received their early training. I am hopeful enough to believe that were the project brought into notice, it would receive sufficient support from nurses and their friends to place it before long on a firm and assured basis. Bournemouth would, I feel sure, be a favourite site for the proposed home. Among the many projects brought forward to commemorate this year, there are few which appear to me more worthy of support than the one now under discussion, and I hope that nurses who are in favour of its establishment will express their views on the subject, so that it may be proved to represent a widely-felt want among the nursing ranks.

### AN OPENING FOR THE PARTIALLY TRAINED.

"ONE WHO DESIRES COMPLETENESS" writes: May I put before you what I feel is a great want? The value of trained nurses to the public is realised by all in dire necessity, but there comes a time when the skill of a trained nurse is no longer necessary—the sick one is through the severity of the illness—the friends begin to breathe freely once more, and then they realise that the sick one is likely to want care for some time to come. The pressure of expense now comes in. I am here speaking of the majority of cases that one has nowadays, either poor gentry or the middle class, who can only just manage the fees. Well, to come to my point. The doctor, the nurse, and the family realise that there is more than any one member of the family can do, for it means a person's whole time and care, and after the anxiety and past strain they are seldom fitted for it. The "want" is a band of women who are conscientious and sympathetic, capable of acting under the doctor's orders, to step in and complete the nurse's work. Many enter hospitals, but sooner or later find that the work is too much, and must be given up. They have gained some knowledge of order, punctuality, cleanliness, &c., &c.; but what becomes of these who just get an insight into the work? They are once more "units" in the world mad to earn a living, call themselves nurse-attendants or nurse-companions, but both they and the public are often deceived in each other. Why should not a club, institution, or office be started to which those having to earn their living and those who need help could meet and be made to "serve one another?" A small remuneration weekly or monthly could be satisfactorily arranged to both. The club (or whatever called) would be a referee for the individual acting as any nursing institution, yet being at the same time a protection to both parties. The co-operative system would, doubtless, answer. Many a good home daughter—a handy girl, who, perhaps, has been looked upon as the nurse in the family—is often obliged to face the world alone, yet has no special accomplishments or attractions, and knows not how to earn her independence. Here is the material wanted—one who would fit in a household, not giving extra work, being one of the family, and making there a home for a certain number of weeks or months, as the case may be. Widows, too, would be glad of such a life. This scheme in plain lines is: (1) A plan to help in finishing a trained nurse's work; (2) a relief to the family responsibility and purse; (3) an acknowledged path by which partly trained people, handy homely girls, or unencumbered widows may serve the public and help each other; (4) remuneration weekly or monthly, say, from 30s. or 40s. per month and upwards, according to the circumstances of the various patients. The writer has recently had no less than six such instances, and heard of much difficulty and trouble in trying to get "someone" after her leaving. This plan she will leave in your hands to take root and flourish, trusting a sympathiser will be raised up to organise such.

### The Book World for Women and Nurses.

[We invite Correspondence, Criticism, Enquiries, and Notes on Books likely to interest Women and Nurses. Address, Editor, THE HOSPITAL (Nurses' Book World), 28 & 29, Southampton Street, Strand, London, W.C.]

THE MATRON'S COURSE: AN INTRODUCTION TO HOSPITAL AND PRIVATE NURSING. By MISS S. E. ORME, Lady Superintendent, London Temperance Hospital. Published by the Scientific Press, Limited, 28 and 29, Southampton Street, Strand, W.C. In handy pocket size, bound in cloth. Price 1s.

Miss Orme, in her introduction to hospital and private nursing, has given to the probationer a sound, practical, and common-sense little hand book, which should start her on the right way to an effectual training. Throughout the book presents high ethics—but these are not raised above the possibilities of human nature, and the author justly criticises the people who talk about the noble work of nursing. "Some of the worst nurses I ever met," she says, "used this phraseology most glibly." Miss Orme disapproves of outdoor uniform, and deprecates—rather too strongly, we think, a tired nurse "lying down" in off time. Does she not think a discretionary rest is the saving grace of many a nurse? One of the best chapters in the book is that on disinfection and dusting, for it puts the matter so clearly, that even the newest recruit to hospital ranks may "read as she runs." And to understand disinfection and dusting is in itself an admirable foundation on which the nurse-pupil builds up her education. Miss Orme is an excellent instructress on etiquette, for her suggestions are so kind, and her hints on general conduct are framed so genially, that not even the most susceptible probationer could be hurt. To put the probationer on her pride, to remind her of the *noblesse oblige* attitude towards her patient, and to put such faults as loud conversations in ward-kitchens and want of sympathy before the probationer in so charming a way, is at once the best method of evoking the chivalry and right-mindedness latent in most young women. "Mamma, I don't mind saying 'please' and 'thank you' when you *ask* me to; but I won't say it when I am *ordered* to" is a sentiment expressed on one occasion by a small boy philosopher. And the probationer is sometimes made of much the same stuff. Miss Orme has tactfully avoided the scolding attitude assumed by many writers when teaching the pupil "what not to do," and this reason alone would commend her book. But coupled with her good nature on ethics is a sound professional knowledge, whose outcome shows itself in a hundred and one most valuable nursing hints. The section on food-stuffs might be simplified somewhat, but the chapter on administration of food teems with admirable suggestions. This little book—excellently produced as to type and finish—should be added to the "nursing kit" of every probationer, to whose hospital lessons it will add much that will be extremely valuable. Miss Orme has a quiet sense of humour—as shown in her pleasant little skits on the "dreamy nurse" and the "mincing nurse"—which gives an added charm to the little volume.

THE ROSY CROSS. By MINA SANDEMAN. (London: The Roxburgh Press. 1897.)

"The Rosy Cross" is one of a collection of eight little tales in one-volume form, tastily got up by the above publishing firm. They are for the most part allegories, and the book is dedicated to "All those who love animals, and also to those who strive to gaze beyond earth's misty veil." At the outset the dedicatory note would appear to be of a mixed order, but to the writer's mind "the beyond of earth's misty veil" is closely connected for human beings with their love of animals while on earth. Miss Sandeman's position, as a champion in the cause of dumb animals, commands our respect and admiration, but in various instances throughout her book the method she employs to emphasise her convictions are such as are hardly likely to forward the claims of her cause. For instance, the ruthless persecutor of animals and birds is placed in close conjunction with the vivisectionist. In one of these stories, "The Deluded Ones," this is the case, and here we find the anti-vivisectionist speaking with a vehemence and certainty characteristic of her class. There is much to be done yet, despite the march of civilisation, in furthering the interests of our four-footed friends, and, as we have said, we much respect the writer of "The Rosy Cross" for her effort in this direction, but in all such efforts there are ways and ways, and we are inclined to think a more forcible one would have been for Miss Sandeman to have kept clear of a question which would lend itself better to a scientific than to a psychical treatment. Some of the other tales are pretty, and show signs of an imaginative mind on the authoress's part.

HYGIENE FOR BEGINNERS. By ERNEST SEPTIMUS REYNOLDS, M.D.Lond. 100 Illustrations. (London: Macmillan and Co. 1896. Price 2s. 6d. 235 pages.)

This is a most excellent little book of its kind, and we can commend its use to both students and teachers of elementary hygiene. It is an enlarged and revised edition of a primer written in 1894 for those attending courses of hygiene given in Evening Continuation Classes or University Extension or County Council courses. The first part consists of nine chapters on "Elementary Anatomy and Physiology." These are well illustrated by good reprints of drawings familiar to us in larger text books. The explanations are lucid and to the point and of such a character as is necessary in order to render the teaching of hygiene intelligible to the beginner. The second part contains nine chapters on "Hygiene." These are written in a clear and readable manner, and the simple diagrams, especially those referring to the heating, airing, and draining of houses, add to the reader's pleasure in no small degree. The quantity of subject matter is surprising considering the size of the book, but if the book were twice the size we could enjoy reading it. We trust it may have the appreciation and circulation it undoubtedly deserves.

### Where to Go.

NIAGARA HALL, S.W.—The Baroness Burdett-Coutts has consented to open the tenth Universal Cookery and Food Exhibition at Niagara Hall on Wednesday, May 12th. Food has an intimate relation with both health and disease, and nurses are every year showing a growing interest in all that appertains to its preparation.

TRAINED NURSES' CLUB.—A lecture will be given by Miss Jane E. Harrison, LL.D., on "Hospital Life in Ancient Greece," in aid of the funds of this club, at 19, Cadogan Square, S.W., on Thursday, May 20th, at half-past five p.m. The lecture will be illustrated from Greek sculpture. Tickets may be had from The Lady Mary Egerton, 19, Cadogan Square, S.W.; Lady Fitzwygram, 92, Eaton Square; or from the hon. sec. of the club, Miss Mary Toynbee, 12, Buckingham Street, Strand, W.C.

THE VICTORIA COMMEMORATION CLUB, 29, SOUTHAMPTON STREET, STRAND.—The Secretary asks us to state that on Saturday, May 8th, there will be a club dinner at half-past six p.m., and again on Wednesday, May 12th, at the same time. Members wishing to reserve a table must write beforehand to the Secretary. Club luncheons are served from half-past twelve to two o'clock, at which for 1s. members can obtain cold meals and salad, with biscuits, cheese, and butter. Nurses will be liable to an entrance fee of a guinea after this month, in addition to their half-year's subscription. Forms of application to be had from the Secretary. On Tuesday next, at three o'clock, Dr. Maclean gives the last of his lectures on "Gynæcological Nursing," and on Wednesday, at three o'clock, Miss Cummings delivers free a lecture on "Hygiene."

### For Reading to the Sick.

### LIVING FOR OTHERS.

"LOVE one another."

**Verses.**

Thy love
Shall chant its own beatitudes,
After its own life working. A child-kiss
Set on thy sighing lips shall make thee glad;
A poor man served by thee shall make thee rich;
A sick man helped by thee shall make thee strong;
Thou shalt be served thyself by every sense
Of service which thou renderest.
—*E. B. Browning.*

That best portion of a good man's life,
His little, nameless, unremembered acts
Of kindness and of love. —*Wordsworth.*

Wouldst thou be happy? Take an easy way.
Think of those around thee, live for them all day;
Think of their pain, their grief, their loss, their care,
All that they have to do, or feel, or bear;
Think of their pleasure, of their good, their gain;
Think of those around thee, 'twill not be in vain.
Live, there are those around, needing thy care,
Pray, there is One at hand, helping thy prayer,
Fight for the love of God, not for renown;
Strive, but in His great strength, not in thine own.

Talk not of wasted affection, affection never was wasted;
If it enrich not the heart of another, its waters returning,
Back to their springs, like the rain, shall fill them full of refreshment. —*Longfellow.*

I see a spirit by thy side, purple-winged, and eagle-eyed,
Looking like a heavenly guide.
Though he seem so bright and fair, ere thou trust his proferred care,
Pause a little and beware!
If he bid thee dwell apart, tending some ideal smart
In a sick and coward heart,
In self-worship wrapped alone, dreaming thy poor griefs are grown
More than other men have known;
Dwelling in some cloudy sphere, though God's work is waiting here,
And God deigneth to be near. . .
If a simple, humble heart seem to thee a meaner part,
Than thy noblest aim and art . . .
Though his words seem true and wise, Soul, I say to thee, Arise!
He is a Demon in disguise. —*A. Proctor.*

**Reading.**

Doing nothing for others is the undoing of oneself. We must be purposely kind and generous, or we miss the best part of existence. The heart that goes out of itself gets large and full of joy. This is the great secret of the inner life. We do ourselves the most good doing something for others.—*Horace Mann.*

We are our best when we try to be it not for ourselves alone, but for our brethren; and we take God's gifts most completely when we realise that He sends them to us for the benefit of other men who stand beyond us needing them.—*Phillips Brooks.*

We may, if we choose, make the worst of one another. Everyone has his weak points; everyone has his faults; we may make the worst of these; we may fix our attention constantly upon these. But we may also make the best of one another. We may forgive, even as we hope to be forgiven. We may put ourselves in the place of others, and ask what we should wish to be done to us, and thought of us, were we in their place. By loving whatever is lovable in those around us, love will flow back from them to us, and life will become a pleasure, instead of a pain; and earth will become like heaven; and we shall become not unworthy followers of Him, Whose name is Love.—*A. P. Stanley.*

### Notes and Queries.

**The contents of the Editor's Letter-box have now reached such unwieldy proportions that it has become necessary to establish a hard and fast rule regarding Answers to Correspondents. In future, all questions requiring replies will continue to be answered in this column without any fee. If an answer is required by letter, a fee of half-a-crown must be enclosed with the note containing the enquiry. We are always pleased to help our numerous correspondents to the fullest extent, and we can trust them to sympathise in the overwhelming amount of writing which makes the new rules a necessity. Every communication must be accompanied by the writer's name and address, otherwise it will receive no attention.**

*Friedenheim.*

(236) Please tell me the address of the free home for the dying called Friedenheim, and if a lady with several years' hospital experience who can give a few hours a day would be of any use or assistance to patients or nurses?—*Nurse Ethel.*

The address is Friedenheim Home of Peace, Sunnyside, Upper Avenue Road, Swiss Cottage, N.W. Write to Miss Davidson, the hon. superintendent. She will probably be very glad to avail herself of any help you may have to offer.

*Address Wanted.*

(237) Can you tell me the address of Miss Hall Hall, who wrote in THE HOSPITAL of May 9th, 1896, about a convalescent home?—*Miss C.*

Miss Hall Hall's address is Northcote, Witley, Godalming.

*Training.*

(238) I am anxious to train as a nurse, and should like to know if a three years' certificate from one of the smaller London hospitals, such as the West London, would be as valuable as one from a provincial hospital with double the number of beds.—*Would-be Pro.*

The value of a certificate depends upon the reputation of the training school which confers it, and the training to be obtained at some provincial hospitals is fully as good as any to be had in London institutions. At the same time a certificate from one of the larger and well-known London hospitals doubtless carries weight, and if you aim at good appointments in the future you might be well advised to apply for a vacancy at one of these.

(239) I want to enter a general hospital as probationer, but find I am too young at present (between 19 and 20). Could I go into some convalescent home until I am older?—*D. B.*

Apply at the Alexandra Hospital for Children, Queen Square, or the East London Hospital for Children, Shadwell. Many children's hospitals take probationers at twenty or even younger. In "How to Become a Nurse" (Scientific Press, 28 and 29, Southampton Street, Strand), you will find a list of institutions, with the ages at which probationers are accepted.

*A District Nurse's Basket.*

(240) Can you tell me where I can buy a basket to use instead of a bag for district work, similar to those used by the Queen's Jubilee Nurses?—*Nurse N.*

Baskets are not used by Queen's Nurses except, we believe, in one district. Do you want a basket for using with a bicycle? If so, your best plan would be to obtain one of those specially made for carrying parcels on a bicycle, and fit it up yourself for your own use. A good suggestion we have just heard is to have a *tin* receptacle made, like a fishing basket in shape, lined and fitted according to requirements. This would be more weatherproof than a basket, and not heavy.

*Asylum News.*

(241) Where can I get the paper called *Asylum News*, mentioned in a recent letter to THE HOSPITAL?—*R. C.*

From Dr. Shuttleworth, Ancaster House, Richmond Hill. Dr. Shuttleworth is hon. secretary of the Association of Asylum Workers.

*Midwifery Training.*

(242) I am a trained medical and surgical nurse, and have a knowledge of midwifery. Where can I get a short term of midwifery training, and obtain a certificate at small expense?—*Enquirer.*

Write to the secretary of the Midwives' Institute and Trained Nurses' Club, 12, Buckingham Street, Strand, London, W.C.

*L.O.S. Examination.*

(243) Please tell me if the names of the successful candidates at the L.O.S. examination in April have been published, and, if so, in what paper?—*M. B.*

You will find the list of successful candidates in the current number of *Nursing Notes* (12, Buckingham Street, Strand, price 2d.)

*A Broken Engagement.*

(244) "Nurse E." wishes to know if the nurse who asked advice in this column on April 3rd as to recovering her fees in a monthly case in which the engagement was broken by the employer, will let her know, either by letter (care of the Editor), or through THE HOSPITAL, what action she finally took and its result.

*Massage.*

(245) Where can a masseur get lessons in bandaging, bed making, lifting helpless patients, and other nursing details? Are there any bandaging classes for nurses or masseuses?—*Masseuse.*

You should go through a course of "First Aid." There are courses or classes on bandaging given at the Trained Nurses' Club, 12, Buckingham Street, in connection with the Society of Trained Masseuses, but we doubt if instruction could be given to masseurs. You might ask the secretary and she might recommend a teacher.

